# A Comparison of the Social-Adaptive Perspective and Functionalist Perspective on Guilt and Shame

**DOI:** 10.3390/bs7040083

**Published:** 2017-12-11

**Authors:** Heidi L. Dempsey

**Affiliations:** Department of Psychology, Jacksonville State University, Jacksonville, AL 36265, USA; hdempsey@jsu.edu; Tel.: +1-256-782-5895

**Keywords:** guilt, shame, emotion, Test of Self-Conscious Affect, TOSCA, social emotions, moral emotions, self-conscious emotions

## Abstract

Within the field of guilt and shame two competing perspectives have been advanced. The first, the social-adaptive perspective, proposes that guilt is an inherently adaptive emotion and shame is an inherently maladaptive emotion. Thus, those interested in moral character development and psychopathology should work to increase an individual’s guilt-proneness and decrease an individual’s shame-proneness. The functionalist perspective, in contrast, argues that both guilt and shame can serve a person adaptively or maladaptively—depending on the situational appropriateness, duration, intensity, and so forth. This paper reviews the research conducted supporting both positions; critiques some issues with the most widely used guilt- and shame-proneness measure in the social-adaptive research (the TOSCA) and discusses the differences in results found when assessing guilt and shame at the state versus trait level. The conclusion drawn is that although there is broad support for the functionalist perspective across a wide variety of state and trait guilt/shame studies, the functionalist perspective does not yet have the wealth of data supporting it that has been generated by the social-adaptive perspective using the TOSCA. Thus, before a dominant perspective can be identified, researchers need to (1) do more research assessing how the social-adaptive perspective compares to the functionalist perspective at the state level and (2) do more trait research within the functionalist perspective to compare functionalist guilt- and shame-proneness measures with the TOSCA.

## 1. Introduction

The systematic study of guilt and shame in the current era began with Darwin’s observations of his young son William and were later published in his comprehensive treatise *The Expression of the Emotions in Man and Animals* [1]. Although Darwin did not clearly distinguish guilt from shame, he did note that shame has a unique behavioral expression. That is, “persons who feel shame for some moral delinquency, are apt to avert, bend down, or hide their faces, independently of any thought about their personal appearance” [1] (p. 157). After Darwin’s insightful exploration of emotional expression, the majority of emotion researchers turned away from research on emotional expression and instead sought to describe the structure of the emotional experience [2]. One notable exception to this trend was within the clinical domain where psychoanalysts focused on examining the role of emotion, particularly guilt, in psychopathology.

Within the psychoanalytic tradition, guilt serves as a self-punisher and excessive guilt was considered to be an important factor in psychological disorders such as neuroticism [3,4,5,6]. Freud postulated that the conscience, or super-ego, develops when the punishing authority of the parents is internalized [7]. Guilt results from violations of commands issued by the conscience, which acts as a self-regulating agency [3]. According to Freud, excessive conscientiousness leads to anxiety, neuroticism, and intense feelings of guilt [8]. Freud gave little credence to shame as a distinct emotion; rather, he saw it as a more primitive version of guilt or a defense mechanism based on a fear of a loss of love, a concept that was extended in Erikson’s theory of psychosocial development [9,10].

Neo-Freudian psychoanalysts continued to see guilt, and not shame, as a dominant emotion in psychological disorders until H. B. Lewis brought shame, the “sleeper in psychopathology” to the forefront. In her seminal work, she sought to differentiate shame from guilt by proposing the now classic distinction, “The experience of shame is directly about the *self*, which is the focus of evaluation. In guilt, the self is not the central object of negative evaluation, but rather the *thing* done or undone is the focus” [11] (p. 30, italics in original). For guilt, the focus is outward, on how the other person is feeling, and thus action tendencies are generally prosocial and other-focused. Consistent with this conceptualization, numerous studies have found that action tendencies for guilt include apologizing, offering to make reparation and amends, vowing to change future behavior, confessing, asking for forgiveness, seeking to restore balance in the relationship, and offering to help others in need [12,13,14,15,16,17,18,19,20]. Shame, in contrast, is associated with avoidance behaviors such as hiding one’s face, collapsing of the body, slumping, or gaze aversion [13,21,22,23]. Shame action tendencies also revolve around expressions of inadequacy, defectiveness, wishing to hide or escape, wanting to save face, and wanting to know that the other person does not view him/her as a lesser person [15,16,18,24,25].

This definitional distinction made by Lewis led to new avenues of research trying to assess the nature of guilt and shame and whether Lewis’s definition had captured an essential distinction between the two emotions. Over the past 40 years, the study of guilt and shame has grown exponentially. One issue with the of field guilt and shame, in particular, but also with the emotion field as a whole, is that there is no agreed upon framework or guiding theory from which researchers can draw their hypotheses, nor are there universally agreed upon measures of the emotions themselves [26]. The current paper reviews two of the main perspectives within the field of guilt and shame and addresses how adoption of each of these perspectives leads to different hypotheses, measurement tools, and ultimately interpretations of results.

## 2. Social-Adaptive and Functionalist Perspectives on Guilt and Shame

The social-adaptive perspective is built on social psychological and clinical theory. In this perspective, guilt focuses the person on the specific action taken or not taken (e.g., “How could I have done *that*?”), while shame focuses the person inward toward the self (e.g., “How could *I* have done that?”) [27]. Because the shame appraisal is global (it involves the entire self), the resulting shame feeling is more intense, painful, and pervasive than is the feeling of guilt. Since a guilt appraisal only involves a specific aspect of the self, the resulting guilt feeling tends to be transitory and localized to a specific deed [28]. Thus, there it is not a specific characteristic of the situation that give rise to a particular emotion, but rather it is the individual’s appraisal of the situation with regard to a self- vs. behavior-focus that differentially gives rise to shame and guilt [29]. Baumeister and colleagues also advocate that guilt should be seen as a fundamentally interpersonal emotion, in contrast to the psychoanalytic perspective of guilt as an intrapsychic phenomenon [12]. Nelissen argues that even self-punishment, as conceptualized by the psychoanalytic theorists, is also part of the inherent adaptivity of guilt, in that, “it may even be the case that self-punishment and prosocial behavior are functionally identical and both actually operate as signals of remorse” [30] (p. 142). Because of the self- vs. behavior-distinction, guilt should be highly correlated to measures of adaptive functioning, such as empathy and prosocial action tendencies and uncorrelated with measures of psychopathology. In contrast, since shame is viewed as so self-critical, it should be highly correlated with both internalizing and externalizing symptoms of psychopathology (e.g., depression, anxiety, hostility, rage, etc.) [31]. Thus, according to Carnì, within the social-adaptive framework, guilt “is perceived as a prosocial phenomenon that has the goal of maintaining, reinforcing, and protecting important interpersonal relationships, particularly with loved ones” [32] (p. 336). In contrast, Tangney and colleagues reason that although shame may have been adaptive at one point in evolutionary history, it is no longer an adaptive or moral emotion [18,31], and “the negative psychological implications of shame are evident across measurement methods, diverse age groups, and populations” [31] (p. 27).

Counter to the social-adaptive perspective, the functionalist perspective of guilt and shame is built on evolutionary and developmental theory [33,34]. Hutcherson and Gross state that the functionalist perspective “argues that emotions are adaptive solutions comprising a coordinated set of appraisals, communicative gestures, physiological responses, and action tendencies tailored to respond to crucial problems faced by our species over the millennia” [35] (p. 720). Consistent with the social-adaptive perspective, the functionalists see emotions as inherently interpersonal because they postulate that they result from the complex interplay between appraisals and behavior, rather than from an intrapsychic source [36]. Campos and colleagues define emotion from a functionalist approach, as “the attempt by the person to establish, maintain, change, or terminate the relation between the person and the environment on matters of significance to the person” [36] (p. 285). For this reason, appraisals, social signals (as reflected appraisals of others), goals, and the degree to which one is making progress toward one’s goals are central to understanding which emotion will be elicited and its accompanying action tendencies [36]. Nelissen, Breugelmans, and Zeelenberg’s [34] (pp. 361–362) evolutionary theory that postulates the ultimate causes of guilt and shame stem from differences in direct and indirect reciprocity appraisals:

At an ultimate level, then, the defining difference between shame and guilt is to be found in the conditions that determine the extent to which they will produce moral behavior. Guilt is more likely to produce moral behavior if conditions favoring direct reciprocity are met—that is, in cases in which there is a likelihood of future encounters with a specific other person who is able to reciprocate with substantial value. Shame is more likely to produce moral behavior if conditions favoring indirect reciprocity are met—that is, in the presence of a relevant audience that witnessed the shameful act. It is further important to realize that from an ultimate perspective, social relationships are nothing more than a resource. Accordingly, any response to threats of concerns for direct and indirect reciprocity needed to be resolved in a cost-efficient manner to the self. So, guilt will often motivate prosocial behavior towards a victim but will do so in a relatively low-cost way, for example, by benefiting nonvictims to a lesser extent rather than incurring more personal costs. Likewise, shame will often motivate norm-compliant behavior (which can be moral) when witnessed by people aware of the shameful act but will be more likely to produce relatively low-cost behavior, such as hiding or leaving a situation (nonmoral) that prevent further reputation loss in the absence of an audience that may enable reputation restoration.

Emotion regulation is also central to the functionalist perspective. To the functionalist, no emotion is inherently adaptive or maladaptive. Rather, adaptivity is determined by emotion regulation processes—how does the individual respond to the emotional experience? In the case of guilt and shame, does the individual work to change the situation or event that caused guilt or shame? Repair the interpersonal relationship that has been damaged? Strive to change others’ impression of him or her? Externalize blame or get angry? Dwell on the emotion? Thus, how one manages and copes with guilt and shame, and subsequently modifies one’s goals and interactions, will determine whether or not these emotions are adaptive or maladaptive for the individual. Cicchetti, Ackerman, and Izard [37] (p. 6, italics in original) state:

Even strong and intense emotions are *not* necessarily maladaptive. Emotions may become maladaptive in two situations. One is when emotions are unconnected to cognitive and affective-cognitive control structures or are connected to cognitive processes and actions that are situationally inappropriate. In the latter case, it is the cognitive appraisal and behavior that are maladaptive, not the emotion. The other situation may involve emotional flooding, where an emotion overwhelms control structures and strategies.

### 2.1. Public-Private Distinction

In contrast to the social-adaptive approach, most functionalist appraisal theories postulate that the difference between guilt and shame lies in the way one thinks one is being perceived by another person. One such appraisal theory suggests that the difference between guilt and shame lies in the *public-private* distinction [38,39]. Within this approach, shame is said to be the more public emotion which is elicited when one’s failures or shortcomings are known or witnessed by others [40]. This is consistent with Cooley’s looking glass self in which shame involves “condemning oneself … but adds the social component, imagining correctly or incorrectly a negative view of self by others” [41] (p. 116). Guilt, on the other hand, is the more private emotion where one fails to live up to one’s own internalized standards or norms, consistent with the psychoanalytic conceptualization of guilt [42]. Smith and colleagues found that, “public exposure of both moral (transgressions) and nonmoral (incompetence) experiences was associated more with shame than with guilt” [38] (p. 138). This appraisal theory is consistent with the evolutionary theories of shame that state that its primary purpose is to maintain social hierarchies and ranks. That is, an individual will feel shame when they accept the negative evaluation of a higher status group member and this will cause the person to either respond with an angry defense strategy or a deference strategy [43]. In fact, Elison and colleagues believe “that many instances of aggression would be better understood as reactions to shame” [44] (p. 448). Conversely, higher status individuals are less likely to express shame [45]. Thus, within an evolutionary framework, shame reminds the individual of his or her relative social rank, lets other group members publically witness that the individual is aware of the violation of moral norms, and prompts submissive, appeasement behavior among lower status group members [34,46,47,48]. In her proposal that shame serves as a general sociometer, de Hooge goes one step further by proposing that “people experiencing shame not only appease to group norms due to their public display of shame, but also *actively engage* in affiliated behaviors that improve their inclusionary status among desired others or groups” [49] (p. 101). In support of this, a recent study by Hejdenberg and Andrews demonstrated that shame and anger were linked only in situations involving criticism and put-downs and not due to a general angry temperament [50]. This is also supported by the finding that shame is directly linked to ostracism and social rejection [51]. Eisenberger and colleagues have also shown that social pain, related to social rejection or exclusion, shares the same neurobiological mechanisms as physical pain [52,53]. Eisenberger suggests that, “over the course of mammalian history, the physical pain system was co-opted by the social attachment system, using pain signals to regulate social relationships” [53] (p. 204). This suggests that public social exclusion or devaluation by relationship partners will not just result in a short-lived emotional experience, but also a feeling of social pain on par with that of physical pain. These findings have led some researchers to argue that shame is a basic emotion and guilt is not even an emotion at all [54,55]. This idea is also consistent with the research showing that while shame does have a recognizable facial expression which incorporates the characteristics of gaze aversion and submissiveness, guilt does not appear to have a unique facial expression [23,56,57].

### 2.2. Trait Guilt and Shame: Social-Adaptive Measures

There have been a wide range of measures used to assess guilt and shame over the past few decades. Although there were measures of guilt developed prior to 1990 (e.g., Mosher’s guilt scales), most were derived using only a psychoanalytic perspective, and thus are not germane to the current review [58,59]. One of the most commonly used measures in the field today is the Test of Self-Conscious Affect (TOSCA). One of the main reasons that the TOSCA is so widely used is because it is available in English versions for children, adolescents, college students, adults, and inmates and has been translated in to multiple other languages [60,61,62,63,64,65,66,67,68,69]. The TOSCA attempts to measure an individual’s guilt- and shame-proneness by asking respondents to imagine themselves in a series of hypothetical scenarios. These situations generally involve some sort of interpersonal moral transgression (e.g., forgetting a lunch date with a friend; making a mistake at work and finding out a coworker is blamed for the error). After the participants read each scenario, they are asked how likely it is that that they would respond in a variety of ways precoded to represent guilt, shame, pride, externalization, and detachment. The TOSCA scenarios were chosen without any specific situational characteristics in mind, other than that the situations were moral in nature. Because the TOSCA was developed within the social-adaptive framework, the TOSCA responses were designed to maximize the self- versus behavior-appraisal. They also tend to portray guilt as an inherently adaptive response and shame as an inherently maladaptive response to the situation. For example, a typical shame response is, “You would feel small … like a rat” and a typical guilt response is “You’d think you should make it up to him as soon as possible.” Although the TOSCA does have many advantages, such as not requiring participants to have a sophisticated vocabulary (they do not have to distinguish between the words “shame” and “guilt”), providing respondents a context in which to project their guilt and shame reactions, and having a variety of questionnaires available for a range of populations, it does have some potential limitations.

One issue with the TOSCA scenarios concerns whether they *equally* elicit shame and guilt responses. Ferguson and colleagues suggested that the TOSCA is biased toward guilt, because participants are clearly causing interpersonal harm in the majority of situations, and thus endorsement of guilt in these scenarios is suggestive of adaptive functioning (as the correlations with symptoms of psychopathology would support) [70,71]. That is, the hypothetical scenarios the TOSCA uses portray the person as having done or not done something which he or she *should* feel guilty about so there is a match between the appraisal of *being* guilty and *feeling* guilty (as is the case in the majority of moral transgression situations). If these scenarios truly are biased more for guilt than shame and a participant endorses shame frequently across these situations, this would indicate a mismatch in appraisal and would suggest maladaptive functioning (as the correlations with TOSCA shame and psychopathology would suggest). Furthermore, because the TOSCA purposefully excludes nonmoral situations, in which shame is the dominant emotion, it is more likely to underestimate people’s tendency to respond with shame.

Additionally, because the TOSCA only uses situations in which the majority of people are likely to report feeling ashamed and especially guilty, this measure does little to tell us about people who have particular problems with the emotion. A useful way to conceptualize this issue is to place it within Kelley’s covariation theory framework [71,72]. One obvious way to assess proneness is to give respondents situations in which the majority of people would feel guilty or ashamed (high consensus). This is the approach the TOSCA takes. However, according to Kelley, to make a true *dispositional* attribution (that is to be able to determine it is something about the person that is causing the behavior as opposed to something about the situation or circumstance), then we should look for the guilt or shame behavior in *low* consensus situations in conjunction with *low* distinctiveness (responding in the same manner in similar situations) and *high* consistency (responding the same way each time he/she is confronted with the same type of situation) [72]. This means the person who is truly guilt- or shame-prone will respond with guilt or shame in a variety of situations, including situations in which the majority of other respondents would not feel these emotions. Thus, the TOSCA is arguably limited because it can only garner information from high consensus situations and, for guilt especially, it is very likely to suffer from ceiling effects.

Beyond the problems with the TOSCA situations, there is also some concern regarding the TOSCA responses. The first issue is regarding the content of the items. Because the TOSCA was developed within the social-adaptive framework, the guilt items represent constructive, interpersonally focused responses to situations (such as trying to make amends and atoning for wrongdoing) and the shame items represent negative, self-derogatory responses and self-directed hostility. For example, several authors have classified the TOSCA-guilt responses into categories. According to Buss, the TOSCA-guilt responses reflect four categories: atoning for or remedying the situation, interpersonal concern, behaving better, and negative but circumscribed self-reactions [73]. The majority of the responses fall within the first category. Luyten, Fontaine, and Corveleyn report the following categories: tendency to repair (5 items), remorse or regret (5 items), negative feelings and a tendency to repair (2 items), and several categories represented by a single item [74]. Surprisingly, there are no items that reflect guilt prompting a desire to confess, a theme that is dominant in much of the literature on guilt [14,75,76,77,78,79]. Also surprising is that there is a TOSCA item that represents expectation for punishment, “I deserve to be reprimanded,” which hearkens back to the psychoanalytic view of guilt and is not commonly represented in the social-adaptive approach [74]. Turning to shame, Luyten and colleagues showed that TOSCA shame items only refer to maladaptive aspects of this emotion: negative self-esteem (nine items), desire to hide or escape (four items), negative emotion (one item), and negative self-appraisal (one item) [74].

One reason the TOSCA authors have used correlate-based judgments (e.g., thoughts, feelings, and behaviors associated with guilt and shame) as the responses in the TOSCA measures is that they have argued that it is too difficult for participants to differentiate between the words *guilt* and *shame* [65,80]. For example, in a scenario that depicts a person as breaking something at work and then hiding it, the respondent is asked to rate the likelihood of responding with guilt, which is depicted as, “You would think: ‘This is making me anxious. I need to either fix it or get someone else to.’” The shame response in the same scenario is, “You would think about quitting.” However, there are several issues with using correlate-based judgments that should be addressed. First, when an individual is making a correlate-based judgment as representative of the emotion, the researcher is making the assumption that there is a near one-to-one correspondence between the judgment and the underlying emotion. This is a risky assumption, given there are many factors that may influence the endorsement of a correlate. For example, social rules that govern behavior operantly condition people to apologize when another is offended in order to smooth interpersonal relations. This apology, though, is not always rooted in a true sense of remorse and does not even necessarily mean that the individual agrees that he or she has caused any harm [81]. In fact, Kugler and Jones showed that TOSCA-guilt was more strongly correlated with awareness of moral standards than it was with the affective experience of guilt [82]. Furthermore, Ferguson and Crowley conducted a confirmatory factor analysis trying to determine the relationship between the most common measures of guilt and shame [70]. They found that although the TOSCA-guilt items load on their own factor, they do not load with other commonly used measures of guilt-proneness. Additionally, once method variance was accounted for (22%), 0% of the guilt variance was accounted for by the emotion trait factor of guilt. Seventy-eight percent of the variance was unexplained by either method variance or the latent factor of guilt. Thus, the TOSCA-guilt items shared no variance in common with other measures of the construct.

A second problem with correlate-based responses is that an individual may feel the underlying emotion but deny that he or she would be likely to respond in the manner given to him/her as an option on the TOSCA. For example, in the scenario presented above, the respondent may think he or she would feel intense shame to the point of hiding in his/her cubicle but not specifically think about quitting, so he or she may indicate there is a very small likelihood that he or she would respond in that manner. Similarly, the individual may think that the guilt response presented in the TOSCA is inappropriate and his or her preferred response in the situation would be to confess that he or she broke the item.

Third, although several authors have suggested that participants cannot reliably distinguish the terms *guilt* from *shame*, which is why the TOSCA uses correlate-based judgments, many other studies of guilt and shame have shown that participants can reliably distinguish these two emotion terms [38,80,83,84]. Ferguson, Stegge, and Damhuis first illustrated children as young as 11 years of age reliably associated having violated a moral norm with the term *guilt* and having committed a social blunder with the term *shame* [21]. Similarly, fifth graders could differentiate characteristics associated with shame from characteristics associated with guilt [21]. Olthof and colleagues investigated whether children’s ratings were different based on whether the response was a correlate-based response precoded to represent shame or guilt or the emotion words of *shame* and *guilt*. They found few differences between the correlate-based ratings and the emotion word ratings [85]. In Olthof and colleagues’ conclusions they argue for the use of emotion word ratings over correlate-based ratings. Because they found correlate-based ratings to be lower than the emotion word ratings, they reasoned that correlate-based ratings may underestimate the intensity of a child’s emotional response. In sum, they stated that, “The data indicate that from the age of 9 upward, children are perfectly well able to differentiate shame from guilt, even when giving term-based judgments” [85] (p. 62). In conclusion, although the TOSCA has been widely used, there are a number of reasons to suggest that it, and the social-adaptive perspective on which it is based, is a restrictive view of guilt and shame.

### 2.3. Trait Guilt and Shame: Functionalist Measures

There have been numerous measures developed to assess trait guilt and shame within the functionalist framework. These measures have generally fallen into two categories: checklist measures and scenario-based measures. The checklist measures have been used nearly as often as the TOSCA and have most often been contrasted with the TOSCA in the literature. Harder and Zalma’s Personal Feelings Questionnaire is a 16-item questionnaire (10 shame; 6 guilt) which asks participants to rate how frequently they feel various emotions such as “mild guilt,” “self-conscious,” “remorse,” or “feel stupid” [86]. In Tignor and Colvin’s recent meta-analysis, they found that check-list type measures differed quite substantially from the TOSCA in their representation of guilt [87]. TOSCA-guilt was positively correlated with prosocial orientation, but there was no correlation between checklist measures of guilt and prosocial orientation. These results are not surprising, given that the majority of checklist measures are designed to measure the frequency of emotion (e.g., the Personal Feelings Questionnaire; PFQ), guilt related to harming others (the Interpersonal Guilt Questionnaire), how much guilt one normally feels (the Guilt Inventory), and so forth [82,83,88]. These measures are more likely to tap guilt emotions that are not well-regulated.

The scenario-based measures that have been developed using the functionalist approach generally include either (1) scenarios in which guilt or shame should not be normative response or (2) responses that reflect problems with emotion regulation. With regard to guilt specifically, Ferguson makes a clear distinction between the state of “being” guilty and “feeling” guilty, which is echoed in dictionary definitions of the term [89]. According to Ferguson, guilt does serve an individual adaptively when there is a match between those situations in which an individual *is guilty* and *feels guilty* [89]. Conversely, guilt is likely to be maladaptive when an individual’s sense of *feeling guilty* does not match his/her actual state of *being guilty*. In these cases, a sense of guilt is misplaced or overexaggerated (e.g., a very young child feels responsible for “causing” his mother’s depression; a woman feels guilty for “allowing” herself to be beaten; a child feels guilty for being more popular than her friend) [90,91,92,93,94].

Olthof’s appraisal theory suggests that a researcher could predict the degree to which a scenario might elicit shame or guilt based on the degree to which two factors were present. The first is whether the scenario leads to the actor’s perceiving that he or she has an unwanted identity (being seen in a way that one does not wish to be seen or is identity-threatening), which would lead to shame. The second is whether the actor perceives that he or she has caused harm to another (hurting a valued relationship partner), which would lead to guilt [95]. This is similar to de Hooge’s recent theorizing in which she sees shame as a general sociometer of inclusive fitness in social groups (see Section 2.1) and guilt as a “specific sociometer, indicating people’s inclusion in one specific relationship (namely, the relationship with the victim)” [49] (p. 102). For example, Olthof et al. and Ferguson et al. independently found scenarios that presented an unwanted identity, without causing harm to another individual, elicited high levels of shame and low levels of guilt in participants [85,96]. Specifically, Ferguson and colleagues generated several scenarios that violated the male gender-role (e.g., getting a flat tire and not being able to change it; crying during an emotional TV commercial in front of friends; being told by a career counselor that one would make a good day-care worker or nurse) [96]. Consistent with the idea that these scenarios would create a stronger unwanted identity for men than women, they found that men reported greater unwanted identities and more intense shame than women did. This research also revealed that the majority of scenarios which present an unwanted identity in the absence of causing harm are nonmoral in nature. When specifically examining nonmoral situations in which an unwanted identity was present, Olthof and associates found that shame was significantly higher than guilt across all ages in their study (i.e., 7- to 16-year-olds) [97].

In addition to developing scenarios that present participants with specific unwanted identities, Olthof also proposed that a researcher could develop scenarios in which interpersonal harm would occur and which would not imply an unwanted identity (e.g., leaving a crying child alone in a hospital to receive his/her treatment; a police officer or soldier having to kill a person in the line of duty) [95]. However, these types of scenarios are likely to be more rare because they require a complete justification of the harm caused, so there is no way the individual can conceive that others are seeing him/her in a negative light. When Olthof examined children’s ratings of guilt and shame in these type of situations (moral wrong but no unwanted identity), he found that children over the age of 12 responded with significantly more guilt in these situations [97]. These results suggest that children must have a very complete understanding of mitigation of an unwanted identity before guilt alone, without the presence of shame, can be experienced.

Although, as Olthof’s appraisal theory suggests, it is possible to find situations in which guilt is likely to occur without a glimmer of shame and shame is likely to occur without a faint trace of guilt, it is likely that these types of situations are not the norm. Rather, the majority of situations one is likely to encounter are moral in nature (with the possibility of causing harm to another) which may, in turn, give rise to an unwanted identity. Because of this overlap, most situations are capable of eliciting both shame and guilt to some degree [10,38,42,98]. The key, though, is understanding that these variables are continuous and that it is very possible to have a situation in which a person is only slightly harmed or inconvenienced (lower levels of guilt), or a situation that may cause lasting damage to the relationship (higher levels of guilt). The same is the case with situations that present unwanted identities to various degrees—some identities may be more “unwanted” than others. Understanding the complex interplay of these variables should allow researchers to create scenarios that are very focused in terms of whether guilt or shame should be the dominant emotion (or whether they should be equally likely to be elicited). For example, the Shame and Guilt Inventory (SAGI) is one such trait measure that has been developed using Olthof’s framework [99].

Mounting empirical evidence has supported the idea that the appropriateness of the emotion to the situation affects the adaptivity of the emotion [21,90]. Ferguson and colleagues demonstrated with children that the context in which guilt occurs has a strong relationship with symptoms of psychopathology [91]. That is, reporting of guilt in scenarios in which guilt is the consensual response (scenarios pilot tested and selected on the basis that the majority of children said they *would* feel guilt in the given situation) was unrelated to internalizing symptoms, whereas experiencing guilt in nonconsensual situations (scenarios pilot tested and selected on the basis that the majority of children said they *would not* feel guilt in the given situation) was strongly related to internalizing symptoms [91]. In a subsequent study, Ferguson and colleagues found that experiencing guilt in hypothetical nonconsensual situations was an even *stronger* predictor of internalizing symptoms than was experiencing shame in consensual situations [90]. This study is especially important because the measure used in this study conceptualizes guilt as an empathic, interpersonally-oriented response. Yet, responding with this “adaptive” form of guilt is not adaptive when the response is in an inappropriate context.

Ferguson and Crowley have also identified a distinctive form of guilt that they termed ruminative guilt [70]. Ruminative guilt is built upon Nolen-Hoeksema’s view that excessive rumination is maladaptive [100]. Ruminative guilt occurs when an individual continues to relive or replay the transgression over and over in his or her mind, thus failing to engage in appropriate emotion regulation. Additionally, it may be characterized by repeated attempts to make reparation and amends [101]. For example, Ferguson and Crowley modified the TOSCA (their version is called the TOSCA-M) to add an additional response to each scenario, which they proposed reflected ruminative guilt. For example, items included “You cannot apologize enough for forgetting …,” “You would bend over backwards for months to make up for it …,” and “For days you would worry about it, repeatedly trying to think of a way to remedy the situation.” All of these items were very behaviorally focused, as opposed to self-focused, consistent with the TOSCA’s operationalization of guilt. Their results showed that ruminative forms of guilt were correlated with guilt frequency and trait guilt, and not correlated with the traditional TOSCA-guilt subscale.

Finally, other issues that likely play into the relative (mal)adaptivity of guilt or shame are the frequency, intensity, duration, and coping strategy used to reduce the guilt or shame [17,24,100,102,103,104,105]. The combination of these different factors may lead to differing levels of maladaptivity. For example, guilt or shame that is too mild for the given situation may also lead to the choice of an inappropriate coping strategy (e.g., a man who may apologize in an offhanded way to his wife for an affair). Measures such as the Compass of Shame Scale have been specifically designed to assess maladaptive shame regulation styles [106,107].

### 2.4. Trait Guilt, Trait Shame, and Psychopathology

Across numerous studies, trait measures of shame have been positively correlated with a wide variety of symptoms of psychopathology, both with clinical populations and non-clinical populations. For example, TOSCA-shame and PFQ-shame have been shown to be related to depression, anxiety, social anxiety, self-derogation, eating disorders, posttraumatic stress disorder, anger, aggression, alcohol and drug problems (including early drinking), decreased self-forgiveness, and low self-esteem [99,102,108,109,110,111,112,113,114]. TOSCA-shame has also been found to be negatively correlated with measures of adaptive functioning, such as self-esteem and life satisfaction, and uncorrelated with measures of empathy [99,115,116]. Thus, there appears to be broad consensus that a disposition toward feeling frequent shame and shame in response to relatively minor moral transgressions is likely to serve an individual maladaptively.

However, the relationship between guilt and psychopathology is very dependent on the type of measure used. The TOSCA, created within the social-adaptive framework, generally shows small or near-zero correlations with symptoms of psychopathology. For example, TOSCA-guilt has been shown to be unrelated to anger, state anxiety, phobic anxiety, depression, interpersonal sensitivity, self-esteem, suicide ideation, negative self-evaluation, externalizing, and social insecurity [88,108,109,115,117,118,119,120,121]. Although the majority of TOSCA studies show there is no relationship between TOSCA-guilt and maladaptive functioning, there are a few select studies that do show a small positive bivariate correlation (usually *r* < 0.20) between guilt and anxiety, depression, obsessive-compulsive behavior, trait anxiety, state anxiety, phobic anxiety, interpersonal sensitivity, social anxiety, anger arousal, anger held in, and self-directed aggression (in these studies, self-esteem is typically also negatively correlated with guilt) [29,110,115,122,123,124]. The correlations between guilt and psychopathology tend to be even smaller when guilt is partialled for shame (Tangney suggests that partialling shame from guilt gives the researcher a measure of “shame-free guilt” which she contends is a more pure measure of the guilt construct [115]; however, many authors disagree with this partialling technique [34,71,99]). Further, guilt, as measured by the TOSCA, has been shown to be positively related to several measures of adaptive functioning, such as an increased propensity toward self-forgiveness, perspective taking, empathic concern, and fantasy, while only marginally with the flipside of empathy—personal distress [111,115,125]. Also, when the feeling of anger is broken down into its constructive and deconstructive components, only the constructive components, such as discussing the incident with the target and taking direct corrective action, are related to guilt [108,123,126,127]. Finally, TOSCA-guilt was found to be negatively correlated with maladaptive anger tendencies such as direct physical, verbal or symbolic aggression, displaced physical aggression, as well as criminal behavior [108,112,123,126,127]. In sum, the majority of results found using the TOSCA do show support for the notion of guilt as adaptive and not related to lasting ill effects for an individual.

In contrast, researchers looking at correlations between guilt and psychopathology using checklist measures have found guilt to be positively correlated with a wide range of indices of maladaptive functioning and symptoms of psychopathology, similar to shame [92,121,128,129,130,131,132,133,134,135]. This is consistent with the idea that frequent, unregulated guilt is likely maladaptive for an individual. Similarly, Crowley and Ferguson found the ruminative guilt subscale of their modified TOSCA-M to be correlated with symptoms of internalizing disorders such as anxiety and depression, while the standard TOSCA-guilt subscale was uncorrelated [136]. Similarly, in a meta-analysis on the links between shame, guilt, and depression, Kim, Thibodeau, and Jorgensen found that when shame and guilt were conceptualized in the manner of the social-adaptive perspective where shame is partialled for guilt and guilt is partialled for shame, then there is support for the idea that shame is more strongly predictive of depressive symptoms than guilt. However, when one separates out TOSCA-guilt from more pathological forms of guilt, “shame and pathological guilt … are *both* important to depressive symptomatology, and to a roughly equal extent” [137] (p. 86, italics in original).

### 2.5. State Guilt and Shame

Recently those researchers looking at state guilt and shame assessment have also begun questioning the research supporting the social-adaptive perspective. Specifically, it does not appear to be the case that only guilt motivates apology and reparation, nor that only shame motivates withdrawal and avoidance, either when looking at the group level or individual level [49,138,139,140]. First, in the domain of group-based emotions, both collective guilt and collective shame have been found to be associated with prosocial motivations to engage in reparations to the outgroup [138,141]. Guilt had a direct relationship with reparations, while the relationship between shame and reparation was mediated by a reputation management motivation [142]. Brown and Cehajic extended this research to suggest that the driving forces behind the shame-reparation relationship are self-pity and empathy felt toward the victimized group, while the driving force behind the guilt-reparation relationship was empathy toward the victimized group [141].

Second, recent research has focused on situations in which shame serves an adaptive purpose [143]. In the interpersonal domain de Hooge and colleagues have found that situational shame motivates prosocial behavior and acts as an interpersonal commitment device when the shame experienced is directly relevant to the current goal pursuit (endogenous shame), but not when the shame is unrelated to the current goal pursuit (exogenous shame), although see Declerck for a counterexample [144,145]. Further, they have found that shame activates an approach motivation when it appears to be possible, and not too risky, to affirm and restore a positive self-view [146,147]. Along these lines, Leach and Cidam found in their meta-analysis that the single best predictor of an individual having a constructive approach orientation after a shame episode was the degree to which the failure was more reparable [139]. In failure episodes where the failure was less reparable, shame led to an avoidance orientation. Thus, although guilt and shame appear to have similar action tendencies, the motivation for engaging in these action tendencies comes from the unique appraisals that one is making in guilt- and shame-inducing situations.

Third, in situations in which one ought to feel shame, a shame response is likely to be an adaptive one because it helps the individual cope with the situation (e.g., after cheating on one’s spouse) [148]. However, in situations in which shame ought not to be felt (e.g., for having a physical or mental disability), then the experience of shame is likely to serve that person maladaptively [90]. Specifically, when an individual experiences shame after a moral lapse or non-moral failure, it may serve the individual adaptively by interrupting the shame-causing behavior and encouraging the person to adhere to societal norms and codes [149,150,151]. For example, most would agree that it is not maladaptive for an individual to experience shame after murdering an unarmed passerby or beating one’s children and spouse. Consistent with this idea, Tangney et al. recently found that inmates who felt shame for their earlier criminal behavior, and who did not externalize blame onto others, had lower recidivism rates [152]. Unlike guilt, the experience of shame has the powerful ability to alter how a person views himself or herself and can deal a crippling blow to an individual’s self-esteem [150]. Because of the intensity and duration of shame, there are perhaps fewer situations in which the average person should feel ashamed. Or perhaps, as Scheff suggests, shame is simply a taboo emotion in our modern Western culture that we attempt to avoid [41]. In support of his thesis, Scheff reported in his analysis of digital books scanned by Google between 1800 and 2007 “the use of the word shame has decreased threefold during the last 200 years in American and British English, French, Spanish, and German books” [153] (p. 117). Further, not all instances of shame appear to be as detrimental to one’s sense of self as one might think. For example, when looking at narratives of shame episodes in individualistic versus collectivist cultures, Wallbott and Scherer found that shame episodes were relatively less intense and shorter in duration in collectivistic cultures than individualistic ones [154]. This is reflective of some of the sociological theories of shame that come from Scheff and Cooley which propose that there are many instances of “low-visibility” shame that are often hidden from view but are central to society’s functioning [153]. Finally, researchers in the field of morality and moral education make the case that both shame and guilt should be considered central moral emotions, even virtues, that should be instilled in childhood [155,156].

Fourth, as mentioned in Section 2.3, there have been several studies conducted on how experiencing guilt in low consensus situations serves an individual maladaptively. This is even more evident when looking at singular traumatic events that have shaped people’s lives and led to emotion regulation problems, such as those tied to posttraumatic stress disorder, trauma-related guilt, and survivor guilt [88,92,121,157]. Specifically, guilt that arises in each of these situations has stemmed from events where one has relatively little control over the outcome and/or the individual holds no culpability for the negative outcome (e.g., being raped, surviving a war). Further, in some other maladaptive forms of guilt, an individual believes that he or she is responsible for other people’s welfare and general happiness (omnipotent responsibility guilt) or that one feels guilty for pursuing one’s own goals and thus being disloyal to family (separation guilt) [88]. Thus, the degree of responsibility one is perceived to have in a situation (by oneself or by others) predicts intensity of guilt [158].

Fifth, all of the aforementioned studies on the adaptivity of guilt have only looked at dyadic relationships between transgressor and victim. When one extends the social circle out a bit further, de Hooge and colleagues have shown that the guilt dynamics also shift. That is, a guilt-driven concern for the victim may actually lead the transgressor to neglect and disadvantage other social partners [159]. Further, “when a third person intervenes in the dyadic situation between a victim and the transgressor by changing the guilt-inducing situation, guilt feelings, reparative intentions, and prosocial behaviours decrease” [160] (p. 1204).

## 3. Discussion

Although the social-adaptive perspective has been one of the dominant approaches, it seems that many current researchers in the field are moving toward a functionalist, evolutionary perspective on guilt and shame. Instead of trying to ask *if* guilt is adaptive and *if* shame is maladaptive, most now seem to be asking the broader question of *when* do guilt and shame serve adaptive and maladaptive purposes. However, one major stumbling block remains with regard to whether the functionalist approach will become the primary approach advocated within this field, and that has to do with the trait measurement of these emotions. Studies using the TOSCA have been published more often and in more prestigious journals than have studies using many other measures of guilt and shame. Thus, to those unfamiliar with the issues regarding trait guilt and shame assessment, the TOSCA seems like the best measure because shame and guilt are portrayed as separate, distinct, and unambiguous emotions. In fact, Leach [140] (p. 18) has recently noted that:

The prevailing view of shame and guilt [to social-adaptive perspective] appears to be especially useful because shame and guilt are thought to be so *qualitatively* different that they are conceptualized as very much like opposites … [but in fact] shame and guilt are more alike than different … And, consistent with this, contemporary emotion research shows there to be small *quantitative* differences between shame and guilt, rather than the dramatic qualitative differences suggested by conceptualizing them as opposites.

To make a convincing case for the functionalist perspective, more studies need to be published using trait measures of guilt and shame based on the functionalist approach. This would allow for a statistical comparison to be made comparing measures using a meta-analysis. Further, researchers need to continue moving beyond simply looking at trait propensity to feel these emotions into looking at guilt and shame *episodes* in broader cultural and contextual domains [161,162]. For example, researchers are now trying to disentangle exactly when shame will result in prosocial approach responses and when it will result in self-protection responses [138,139,163]. Additionally, research needs to further address Wallbott and Scherer’s findings that shame episodes were relatively less intense and shorter in duration in collectivistic cultures than individualistic ones [153]. For instance, it would be useful to collect narratives from participants from different cultures reporting guilt and shame episodes where they felt the emotion served an adaptive or maladaptive purpose in their lives. Perhaps this will shed more light on the complex interplay between self- and other-appraisal and their relationship to the (mal)adaptive consequences of these emotions. Future research should also continue to look at the definitions of these two emotions in an attempt to clearly separate out instances of shame and instances of guilt. The narrative study, suggested above, would also be useful for examining how participants define these emotions and whether participants see guilt and shame as distinct emotions, the same emotion, or one emotion as subsumed under the other. Finally, guilt and shame are both social emotions which are often assessed in non-social or dyadic situations [164,165]. De Hooge has recently begun to investigate how guilt and shame play out when there are multiple actors present, but more needs to be done in this domain.

In conclusion, there seems to be no clear consensus on which perspective will end up being supported by the majority of emotion researchers. This mainly stems from the fact that the social-adaptive researchers have great depth in the field, but not enough breadth (i.e., many systematic studies, but conducted with a single measure of trait guilt and shame). The functionalist researchers, on the other hand, have great breadth, but not much depth (i.e., a wide range of studies on both state and trait guilt and shame, but not enough programmatic research to yield conclusive results). Thus, in order to fully compare and contrast the two perspectives, researchers need to (1) conduct *more* studies comparing the TOSCA to other measures of guilt and shame and (2) to conduct systematic studies looking at which approach best accounts for emotions, cognitions, and behaviors in a *wide variety* of guilt- and shame-inducing situations.
